# Mutation of the Polygalacturonase Gene *AcoPG3* Deferred Softening of Pineapple Fruit

**DOI:** 10.3390/biology14050474

**Published:** 2025-04-25

**Authors:** Haiyan Shu, Aiping Luan, You Wang, Junhu He, Qing Wei, Rulin Zhan, Shenghe Chang

**Affiliations:** 1 Tropical Crops Genetic Resources Institute, Chinese Academy of Tropical Agricultural Sciences, Haikou 571101, China; shuhy@catas.cn (H.S.); aipingluan@catas.cn (A.L.); wangyouid@gmail.com (Y.W.); hejunhu@catas.cn (J.H.); qingwei_321@163.com (Q.W.); zhanrulin555@163.com (R.Z.); 2 Sanya Research Institute, Chinese Academy of Tropical Agricultural Sciences, Sanya 572025, China; 3 National Key Laboratory for Tropical Crop Breeding, Sanya 572025, China; 4 Key Laboratory of Crop Gene Resources and Germplasm Enhancement in Southern China, Ministry of Agriculture and Rual Affairs, Haikou 571101, China; 5 Key Laboratory of Tropical Crops Germplasm Resources Genetic Improvement and Innovation of Hainan Province, Haikou 571101, China

**Keywords:** cell wall, pectin, shelf life, degradation, mutant

## Abstract

A large number of pineapple (*Ananas comosus*) fruits are discarded in China every year due to softening. However, the underlying molecular mechanism is still unknown. *AcoPG3* (GenBank accession number: XM020243935), a pineapple gene of polygalacturonase, was found to be the major gene responsible for the softening of pineapple fruit. Fruit of *AcoPG3*-overexpressing tomato (*Solanum lycopersicum* var. Jingfan 101) begins to soften 9 days earlier than that transformed with a net vector. Fruit of *AcoPG3*-overexpressing pineapple (APG3-2) begins to soften 6 days earlier than that transformed with a net vector. Fruit of MPG3-1, a pineapple line in which *AcoPG3* is mutated, begins to soften 31 days later than that transformed with a net vector. The sequence of polygalacturonase activities in fruit from the highest to the lowest was APG3-2, wild type, MPG3-1. The same sequences were also found in the liquid content of apoplast and the electrolyte leakage of pineapple pulp. The order of methyl-esterified pectin content in the pulp cell wall, from the highest to the lowest, was MPG3-1, wild type, and APG3-2. The same order was also observed for the contents of non-methyl-esterified homogalacturonan and rhamnogalacturonan-I in the pulp cell wall. The *AcoPG3* mutation resulted in a decrease in polygalacturonase activity in pineapple fruit, decreasing the degradation of methyl-esterified pectin, non-methyl-esterified homogalacturonan, galactan and rhamnogalacturonan-I in the pulp cell wall. Fruit softening can be deferred, and the shelf life can be extended by mutating the *AcoPG3* gene.

## Simple Summary

*AcoPG3* (GenBank accession number: XM020243935), a pineapple gene of polygalacturonase, was found to be the major gene responsible for the softening of pineapple fruit. *AcoPG3* mutation resulted in a decrease in polygalacturonase activity in pineapple fruit, decreasing the degradation of methyl-esterified pectin, non-methyl-esterified homogalacturonan, galactan and the backbone of rhamnogalacturonan-I. The degradation of cell walls and fruit softening can be deferred by mutating the *AcoPG3* gene.

## 1. Introduction

Pineapple (*Ananas comosus*) is a well-known tropical fruit. Around 66,700 hectares of land is planted with pineapple in China every year (Data from Southern Asian Tropical Crops Office). The annual total yield is 1,560,000 tons. Most of the pineapple fruits in China are harvested in April and May, when oranges and litchis are also harvested. A large number of pineapple fruits cannot be sold on time. Pineapple fruit can only be stored for 7–10 days at room temperature. After that, it begins to soften and rot. The amount of pineapple fruits discarded because they were not sold on time in China every year is about 20% of the total yield. It is important for the pineapple industry to study the mechanism underlying the softening of pineapple fruit and to breed new cultivars with a longer shelf life.

The main cause of fruit softening is the degradation of the cell wall [1]. Both enzymatic and nonenzymatic factors can result in the degradation of the cell wall [2]. The increase in the activities of endogenous hydrolases is the main cause for the degradation of the pulp cell wall [3]. The main components of the pulp cell wall include pectin, cellulose and hemicellulose [4]. Pectin is the most abundant component, comprising more than 35% of the total dried cell wall [5]. Pectin includes the smooth region and the trichoid region. The smooth region is made up of homogalacturonan (HG), which is composed of D-GalA residues linked with a glycosidic bond [6]. The quantity of HG is about 65% of pectin in the cell wall [6]. The trichoid region is composed of rhamnogalacturonan-I (RG I) and rhamnogalacturonan-II (RG II) [6]. The backbone of RG I is a dimer of 1,2-α-L-rhamnose-1,4-α-D- galacturonic acid [7]. The lateral chain bonds with a Rha residue [7]. The backbone of RG II is HG. Its lateral chain bonds with a Gal A residue [8]. The endogenous hydrolase in pineapple fruit includes polygalacturonase, pectinesterase (PME), β-galactosidase (GAL), xyloglucan endotransglycosylase (XET) and cellulase (CX) [9]. Polygalacturonase is the main endogenous hydrolase in pineapple fruit. Only minimal activities of pectinesterase and β-galactosidase were found when the cell wall of pineapple pulp was degraded [9]. There was no significant relationship between the degradation of the pineapple cell wall and xyloglucan endotransglycosylase or cellulase [9].

Plant polygalacturonase belongs to glycohydrolase family 28, including exo-polygalacturonase (exoPG), endo-polygalacturonase (endoPG), and rhamnogalacturonan polygalacturonase (RhaPG) [10,11]. The α-1,4 glycosidic bond between galacturonic acid (GalA) in the main chain of pectin is cut randomly by endoPG. GalA in the non-reduced HG backbone is cleaved by exoPG one by one [12]. The GalA-rhamnogalacturonan bond in RG I is cut by RhaPG [11].

Most polygalacturonase genes in plants exist as family members [11]. The pectin content in the root elongation zone of rice transformed with polygalacturonase gene *OsPG2* was less than that in the wild type [13]. Apple trees transformed with *MdPG1* mature earlier than the wild type [14]. The adhesion of the abscission zone in the petiole of apple trees transformed with *MdPG1* decreased [14]. The expressions of strawberry polygalacturonase genes *FaPG1* and *FaPG2* in mature fruit were the highest [15,16,17,18]. After *FaPG1* expression in strawberry was reduced through RNAi, the pectin degradation in *FaPG1*-RNAi fruit was lower than that in control fruit [19,20]. The expression of *FaPG2* in strawberry fruit of the apt-softening cultivar was higher than that in a non-apt-softening cultivar [16]. The contents of HG and RG I pectin in *FaPG1*-RNAi and *FaPG2*-RNAi fruits were higher than those in control fruits [21]. Polygalacturonase plays a major role in strawberry fruit softening [22]. Silencing FxaC_21g15770, a strawberry polygalacturonase gene, could significantly improve the firmness of strawberry fruit without affecting the fruit color, soluble solids, cellulose, and hemi-cellulose [22]. The polygalacturonase-catalyzed solubilization and depolymerization of pectin play a central role in peach fruit softening [23]. Suppressing the expression of polygalacturonase genes *PpPG21* and *PpPG22* in melting flesh peaches significantly reduced the polygalacturonase activity and maintained the fruit firmness during the late shelf-life stage [23].

The aims of this research are as follows: (1) to study the molecular mechanism underlying the softening of pineapple fruit; (2) to find polygalacturonase genes expressed in pineapple fruit; (3) to identify whether these genes have a polygalacturonase function; (4) to study the relationship between the expressions of these genes and the softening of pineapple fruit; (5) to study whether the expressions of these genes affect pectin components in the flesh cell wall; (6) to study whether the expressions of these genes affect the liquid content in pulp apoplast. To the best of our knowledge, this is the first report studying the mechanism underlying the softening of pineapple fruit. These results will lay foundations for breeding pineapple cultivars with a long shelf life.

## 2. Materials and Methods

### 2.1. Materials

Pineapple used in this research was Tainong17, which was planted in experimental field of Haikou Experimental Station, Chinese Academy of Tropical Agricultural Sciences, in Danzhou, Hainan Province, China. All of the chemicals and reagents used in this research were bought from Sigma-Aldrich (St. Louis, MO, USA) or Sangon Biotech (Shanghai, China). All of the plasmids were sequenced in Sangon Biotech (Shanghai, China).

### 2.2. Vector Construction

*AcoPG3*-overexpressing plants and *AcoPG3* mutants were created according to the papers published [24,25]. When *AcoPG3*-overexpressing plant was created, *AcoPG3* (GenBank accession number was XM020243935) and its promoter (from 1 bp advanced the starting codon to 1.6 kb upstream) were cloned from pineapple genome and inserted into pMD-19T. The formed plasmid was named p19T-pro*AcoPG3*-*AcoPG3*. *GusA* was isolated from pBI121 (GenBank accession number AY781296) and inserted into multiple cloning sites of pCAMBIA1300, forming plasmid pCAMBIA1300/GUS. Pro*AcoPG3*-*AcoPG3* was isolated from p19T-pro*AcoPG3*-*AcoPG3* and inserted into pCAMBIA1300/GUS. The formed *AcoPG3*-overexpressing vector was named pCAMBIA1300/GUS-pro*AcoPG3*-*AcoPG3* (pC13GPRPG3).

### 2.3. Pineapple Transformation

Pineapple transformation was performed according to a study [24]. Tainong17 pineapple was transformed using agrobacterium EHA105. GUS activation was measured according to a study [26]. Genomic DNA was extracted from pineapple leaves. PCR was performed using P1 (5′-CTATTTCTTTGCCCTCGGACGAGTG-3′) and P2 (5′-ATGAAAAAGCCTGAACTCACCGCGA- 3′) as primers for identifying the transgenic plants.

### 2.4. Tomato Transformation

Tomato (*Solanum lycopersicum*) cultivar “Jingfan 101” was used for identifying the function of *AcoPG3.* Tomato transformation was performed according to a study [27].

### 2.5. Construction of Pineapple AcoPG3 Mutant Lines

Gene-editing vector was constructed according to method published in [25]. The transition vector used in this research was SK-gRNA, and the Cas9 vector was pC1300-Cas9 [22]. The resulting vector was named pC13Cas9PG3, which was transformed into agrobacterium EHA105. Pineapple embryogenic callus was infected with the transformed agrobacterium. Target sequences of the transgenic plants were amplified and sequenced. Potential off-target sequences were also amplified and sequenced.

### 2.6. Determination of Fruit Firmness

Fruit firmness was measured according to a study [28]. The instrument used was food property analyzer TMS-Pro (FTC, Sterling, VA, USA). The data were collected from fruit base, middle part and far away from fruit base. The average values were used. Six fruits were measured for each treatment.

### 2.7. Polygalacturonase Activity Measurement

Polygalacturonase activity was determined according to a study [29]. Thus, 5 g of frozen pineapple pulp was added to 20 mL extracting buffer (50 mmol/L phosphate sodium buffer (pH6.0), 2.4 mol/L NaCl, 0.5 g PVP, a little quartz sand). The mixture was ground at 4 °C for 2 min. The mixture was centrifuged for 30 min at 10,000 rpm and 4 °C. The supernatant was put in a dialysis bag (MW cut-off12800 cellulose membrane) (Sigma-Aldrich, St. Louis, MO, USA). Dialysis was performed in 50 mmol/L of phosphate sodium buffer (pH4.5) containing 150 mmol/L NaCl for 24 h. The buffer was changed one time during the process. The supernatant was the polygalacturonase crude enzyme. Then, 0.1 mL polygalacturonase crude enzyme was added to 0.4 mL 0.2% pectin solution. The reaction was performed in 40 °C water for 1 h. Further, 1.5 mL DNS (3,5-dinitrosalicylic acid) was added and put into 100 °C water for 10 min. The mixture was cooled to room temperature and diluted to 5 folds. OD value was measured at a wavelength of 540 nm. The standard curve was drawn using galacturonic acid as standard substance. All experiments were repeated three times, and the average data were used.

### 2.8. Extraction of Cell Wall

Pineapple flesh cell wall was extracted according to a study [30]. Pineapple fruit was collected at different stages and was stored in −40 °C freezer. The tissues were ground into powder using liquid nitrogen. Further, 20 mL of PAM (phenol/acetic acid; water = 2:1:1 *w*/*v*/*v*) was added for 10 g powder. The mixture was mixed using glass rod for 10 min at room temperature. Then, 10 mL 90% DMSO was added and mixed for 5 min. The mixture was centrifuged at 4000× *g* 4 °C for 10 min. The supernatant was collected and centrifuged at 10,000× *g* 4 °C for 30 min. The pellet was the cell wall extracts.

### 2.9. Cell Wall Component Analysis

Cell wall component was analyzed using carbohydrate chips, according to a study [31]. Cell wall extract was ground using glass beads in buffer (0.1 M Na_2_CO_3_, 4 M KOH, 31% quadrol prepared using 0.78 M cadmium oxide) for 30 min. The mixture was shaken slowly for 1 h at room temperature. The mixture was centrifuged at 2700× *g* for 15 min at room temperature. The supernatant was the water fraction. The pellet was further analyzed, and Na_2_CO_3_ fraction, KOH fraction, cadoxen fraction were established. Each fraction was diluted 4 times. The samples were pipetted onto nylon membrane using ArrayJet Sprint (ArrayJet, Roslin, UK). Samples were detected using monoclonal antibody. Second antibody was goat anti-rabbit immunoglobulin G. First antibodies and second antibody were bought from PlantProbes (Leeds, UK). Results were scanned using CanoScan 8800F scanner and transformed into TIFF format. The results were analyzed using ImaGene 6.0 microarray analysis software (BioDiscovery, El Segundo, CA, USA).

### 2.10. Liquid Content in Intercellular Space

Liquid contents in apoplast were determined according to a study [32]. Fruit flesh was cut into cubes with 1 cm edge length. Cracked tissues and cells were washed away using ultrapure water. Ten cubes were put into a 50 mL tube. Ultrapure water was added into the tube until the total volume was 20 mL. The liquid was transferred into another tube. The liquid volume was measured and named A. B was the sample volume. B = 20 − A. Samples were centrifuged at 300× *g*, 4 °C for 30 min. The volume of supernatant was named C. C/B was the liquid content in apoplast.

### 2.11. Cell Membrane Permeability Determination

Cell membrane permeability was measured according to a study [33]. Fruit flesh was cut into cubes with 1 cm edge length. The cubes were divided into 3 portions, 1 g for 1 portion. They were put in 3 different beakers. Also, 50 mL denoised water was put into the beaker. The beaker was put into vacuum drier. The air was removed using vacuum air pump for 30 min. Then, air was put into beaker slowly. Beakers were fetched from the drier and subjected to room temperature for 1 h. They were shaken for 1 min, one time every 5 min. After that, flesh cubes were taken out. Electrical conductivity of the solution in the beakers was measured using electric conductivity meter. The average data were used as solution conductivity. The conductivity of distilled water was used as control. Electrolyte relative leakage was used for demonstrating cell membrane permeability. Electrolyte relative leakage (%) = (conductivity of treatment − control conductivity)/(conductivity of boiled solution − control conductivity) × 100.

## 3. Results

### 3.1. Increased Polygalacturonase Activity Is the Main Cause for the Softening of Pineapple Fruit

After pineapple fruits were collected and stored at room temperature, the activities of the main endogenous hydrolases were determined in different stages. The results showed that the polygalacturonase activity in each stage was remarkably higher than the activities of PME, GAL, XET and CX (Table 1). This indicated that polygalacturonase was the main hydrolase responsible for degrading the cell wall of pineapple pulp. During fruit ripening and softening at room temperature, the polygalacturonase activity increased first. On the ninth day, polygalacturonase activity reached the maximum. After that, polygalacturonase activity decreased a little. Polygalacturonase activity in the middle stage and late stage was higher than that in the early stage (Table 1). Water-soluble pectin contents in pulp were positively correlated with polygalacturonase activity (r = 0.8507) (Table 1 and Table 2). Water-soluble pectin contents in pulp were negatively correlated with fruit firmness (r = −0.7440) (Table 1 and Table 2). These demonstrated that polygalacturonase can improve the dissociation of the cell wall of pineapple pulp. Polygalacturonase played a primary role in degrading fruit pectin and fruit softening. Polygalacturonase activity enhancement is the main cause for the degradation of the cell wall of pineapple flesh.

### 3.2. AcoPG3 Was the Main Gene Encoding Polygalacturonase in Pineapple Fruit

**Figure 1:**
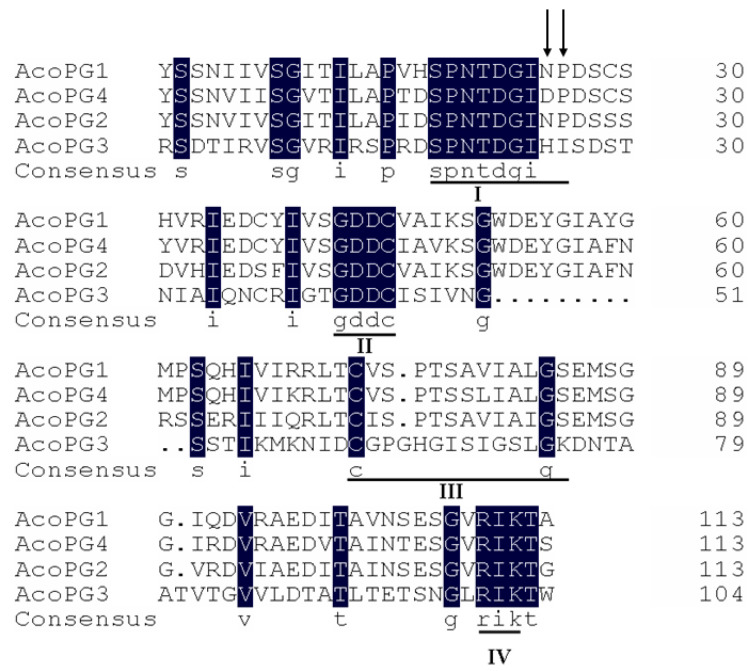
Conserved domains in pineapple polygalacturonase amino acid sequences I to IV represented the conserved domains. The conserved domains were underlined. Two amino acids have been mutated in conserved domain I of AcoPG1, AcoPG2 and AcoPG4. The positions of the amino acids mutated were shown using arrow heads. AcoPG1, AcoPG2 and AcoPG4 had no the conserved domain III. The dark-blue background showed the complete identical amino acids in AcoPGs amino acid sequences.

**Figure 2:**
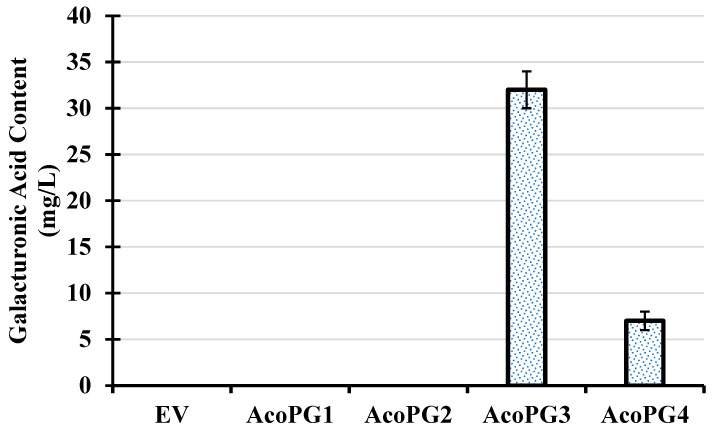
Galacturonic acid content in sonicated pellet mixture of *E. coli* harboring *AcoPG* genes or empty vector. EV represented the recombinant extracted from *E. coli* transformed with net vector. AcoPG1 to AcoPG4 represented recombinant protein extracted from *E. coli* transformed with AcoPG1 to AcoPG4.

There were 32 polygalacturonase genes in the pineapple genome. RT-PCR analysis showed that only four polygalacturonase genes were expressed in fruit. They were named *AcoPG1* (XM020257213), *AcoPG2* (XM020237815), *AcoPG3* (XM020243935), and *AcoPG4* (XM020229423) (Figure 1). They were cloned into a pET28a (+) vector and transformed into *E. coli* BL21 (DE3). The transformed *E. coli* was cultured at 28 °C for 5 h and induced for 0.5 h using 0.3 mmol/L IPTG. The cultures were centrifuged, and the supernatant was discarded. The pellet was sonicated and cultured with 0.5% polygalacturonic acid solution for 0.5 h. 3,5-dinitrosalicylic acid (DNS) was added, and the mixture was put in 100 °C water for 5 min. After the mixture was cooled to room temperature, the light absorption value was measured using an ultraviolet spectrophotometer at 540 nm wavelength. The light absorption value of the mixture containing cultures of *E. coli* harboring a net vector was used as control (EV). The results showed that there was no galacturonic acid in the cultures of AcoPG1, AcoPG2 and EV, demonstrating that polygalacturonic acid in these cultures had not been transformed into galacturonic acid (Figure 2). Proteins encoded by *AcoPG1* and *AcoPG2* had no polygalacturonase functions. Galacturonic acid contents in mixtures containing *E. coli* transformed with *AcoPG3* and *AcoPG4* were much higher than that of EV, indicating that lots of polygalacturonic acid was transformed into galacturonic acid in these two mixtures (Figure 2). *AcoPG3*- and *AcoPG4*-encoded proteins had polygalacturonase functions. However, the polygalacturonase activity of AcoPG3 protein was about 4.6-fold of the AcoPG4 protein, indicating that *AcoPG3* was the main gene responsible for encoding polygalacturonase in pineapple fruit.

### 3.3. Tomato Fruits Transformed with AcoPG3 Began to Soften Earlier than Control

**Figure 3:**
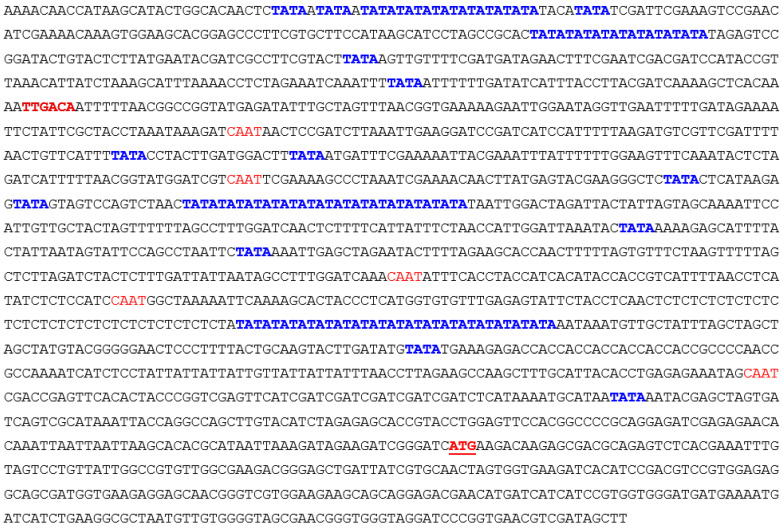
Pineapple *AcoPG3* promoter sequence. TATA was shown in blue bold letter. CAAT sequence was shown in red letter. -35TTGACA sequence was shown in red bold letter. Starting codon was shown in red bold letter and underlined.

**Figure 4:**
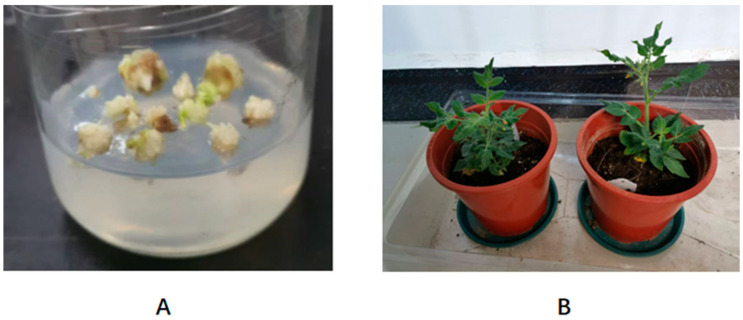
Tomato transformed with *AcoPG3*. (**A**) showed tomato callus transformed with *AcoPG3* cultured on medium containing 20 μg/L hygromycin. (**B**) showed that transgenic tomato seedlings grown in culturing pot containing gardening soil.

**Figure 5:**
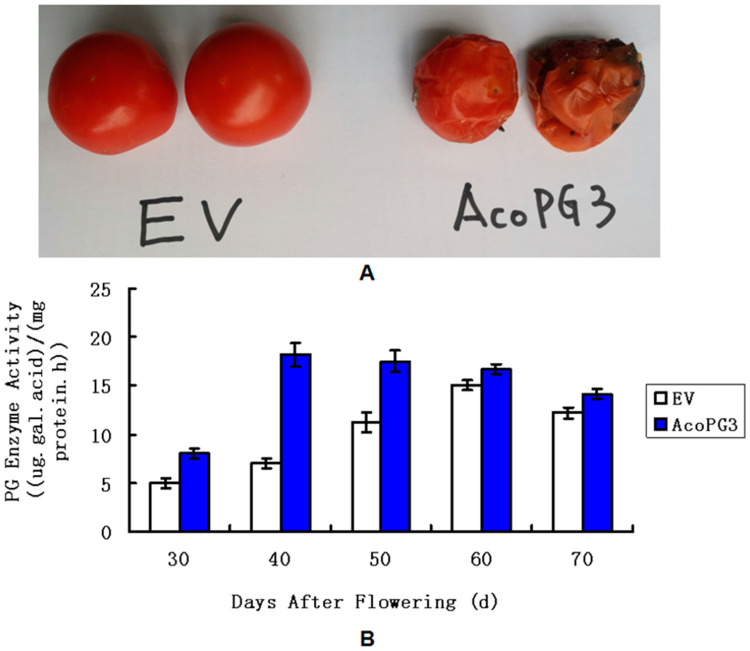
Tomato fruits softening and polygalacturonase activity determined in different time. (**A**) showed the tomatoes 55 days after flower. (**B**) showed polygalacturonase activities of tomato fruits in different stages. EV represented the tomato transformed with empty vector. AcoPG3 represented the tomato transformed with *AcoPG3*.

To identify the function of *AcoPG3* in plants, *AcoPG3* with its promoter (1.6 kb sequence advanced from the starting codon of *AcoPG3*) (Figure 3) was transformed into tomato (*Solanum lycopersicum* var. Jingfan 101). Six seedlings were obtained on regenerated media containing 20 μg/L hygromycin. PCR experiments showed that all six seedlings were transgenic plants. Seeds were collected and germinated. Only the seeds of plant 2 and plant 5 can germinate. Because the plant type of plant 2 was similar to that of the wild type, the seeds of plant 2 were propagated and used in the following experiments (Figure 4). The results showed that fruits of plant 2 begin to soften 9 days earlier than the fruit transformed with a net vector (Figure 5A). At any time, the polygalacturonase activity in fruits transformed with *AcoPG3* was significantly higher than that in fruit transformed with a net vector (Figure 5B). These demonstrated that *AcoPG3* had the function of degrading the cell wall of tomato pulp.

### 3.4. The Softening of Pineapple Fruit Was Regulated by the Expression of AcoPG3

To further identify *AcoPG3*’s function in degrading the cell wall of pineapple pulp, a callus of Tainong17 was infected with Agrobacterium harboring the plasmid pC3300-pro*AcoPG3*-*AcoPG3*. Four seedlings were obtained in regenerated media containing 20 μg/L hygromycin. Using RT-PCR, they were all found to be transformed successfully. They were named APG3-1 to APG3-4. APG3-2 was selected for the following experiments as it was a similar plant type to the wild type. The plant transformed with a net vector was named EV-1.

**Figure 6:**
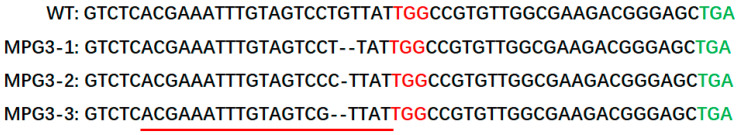
Gene edition sequence of *AcoPG3*. Potential pre-formed stop codons were shown with green and bold capital letters. Target sequence was underlined. PAM site was shown in red and bold capital letters, and possible stop codon were over-lined.

To identify *AcoPG3’s* function comprehensively, *AcoPG3*-mutant pineapple lines were created using the CRISPR/Cas9 system. Seven plants were obtained in a regenerated medium containing 20 μg/L hygromycin. Genomic DNA was extracted from leaves. The target sequence was amplified and sequenced, and three plants were found to be successfully mutated and had no off-target mutation (Figure 6). They were named MPG3-1 to MPG3-3. Two bases were deleted, and a stop codon formed in advance in the target sequence of MPG3-1 (Figure 6). One base was deleted and one base was deleted/inserted in MPG3-2 (Figure 6). Two bases were deleted and one base was deleted/inserted in MPG3-3 (Figure 6). MPG3-1 was selected for the following experiments. The pineapple transformed with a net vector was named EV-2.

APG3-2, EV-1, MPG3-1, EV-2 and WT were planted in a pot containing gardening soil in a greenhouse. When two eyes of the WT fruit became yellow, fruits were collected and stored at room temperature. The results showed that at most time points, the firmness of APG3-2 fruit was the lowest (Table 3). The firmness of MPG3-1 fruit was the highest. The firmness of WT, EV-1 and EV-2 was similar. Even on the 30th day after harvest, the firmness of MPG3-1 was still similar to that of WT fruit on the collection day (Table 3). WT fruit began to soften on the 6th day after harvest, while MPG3-1 fruit began to soften on the 37th day after harvest. APG3-2 fruit began to soften on the collection day. These results demonstrate that overexpressing *AcoPG3* can accelerate the softening process of pineapple fruit. Mutating *AcoPG3* can delay the softening process. The softening of pineapple fruit was regulated by the expression of *AcoPG3*.

### 3.5. AcoPG3 Was the Determinator for Polygalacturonase Activity in Pineapple Fruit

To study the mechanism that *AcoPG3* regulates the softening of pineapple fruit, polygalacturonase activities in pulp of different lines were determined. The results showed that polygalacturonase activities in WT, EV-1, EV-2 begin to increase on the third day (Table 4). On the ninth day, polygalacturonase activities reached the maximum value. After that, polygalacturonase activities decreased. Polygalacturonase activity in APG3-2 fruit on the collection day was the highest. Polygalacturonase activity in APG3-2 fruit decreased with the storage time. Polygalacturonase activity in MPG3-1 fruit always increased. But the increasing velocity was very slow. Even on the 30th day after collection, the polygalacturonase activity of MPG3-1 was still less than that of WT on the collection day. At any time detected, polygalacturonase activity in MPG3-1 fruit was much less than that in APG3-2, WT, EV-1 and EV-2 fruit. At most times detected, polygalacturonase activity in APG3-2 fruit was higher than other pineapple lines. *AcoPG3* was the determinator for polygalacturonase activity in pineapple fruit.

### 3.6. Component Contents in Cell Wall of Pineapple Pulp Were Modified by the Expression of AcoPG3

To study how the expression of *AcoPG3* affects the cell wall of pineapple pulp, using the cell wall of pineapple pulp as materials, experiments of carbohydrate chips were performed. The results showed that in the aqueous phase extracted from the cell wall of APG3-2 pulp, the content of methyl-esterified pectin (recognized by antibody JIM7) was significantly lower than that from MPG3-1, EV-1, EV-2, and WT pulp (Table 5). The methyl-esterified-pectin content in the cell wall of MPG3-1 pulp was higher than that in EV-1, EV-2, and WT pulp. No significant differences were found among methyl-esterified-pectin contents in EV-1, EV-2 and WT pulp.

Non-methyl-esterified HG was recognized by antibody LM18 and LM19. RG I galactose was recognized by antibody LM5. The RG I backbone was recognized by RU1 and RU2. The contents of non-methyl-esterified HG and RG I in the Na_2_CO_3_ fraction extracted from the cell wall of APG3-2 pulp were lower than those extracted from MPG3-1, EV-1, EV-2, and WT pulp (Table 5). Non-methyl-esterified HG and RG I contents in the cell wall of MPG3-1 pulp were higher than those in EV-1, EV-2, and WT pulp, respectively. The contents of non-methyl-esterified HG in the cell walls of EV-1, EV-2 and WT pulp were similar. The same result was also found in RG I contents (Table 5).

The contents of xyloglucan (recognized by LM15 and LM25) and xylan in cell walls of APG3-2, MPG3-1, EV-1, EV-2, WT pulps were similar (Table 5). Mannan was recognized by LM21. LM21 signals in all samples were faint. These demonstrated that overexpressing *AcoPG3* enhances the degradation of methyl-esterified, non-methyl-esterified HG, galactose and the RG I backbone. Overexpressing *AcoPG3* had no roles in degrading mannan, xyloglucan or xylan. Mutating *AcoPG3* decreases the degradation of methyl-esterified pectin, non-methyl-esterified HG, galactose, and the RG I backbone. Mutating *AcoPG3* had no roles in degrading mannan, xyloglucan or xylan.

### 3.7. Liquid Contents in Apoplast and Electrolyte Leakage of Pineapple Pulp Were Regulated by AcoPG3

**Figure 7:**
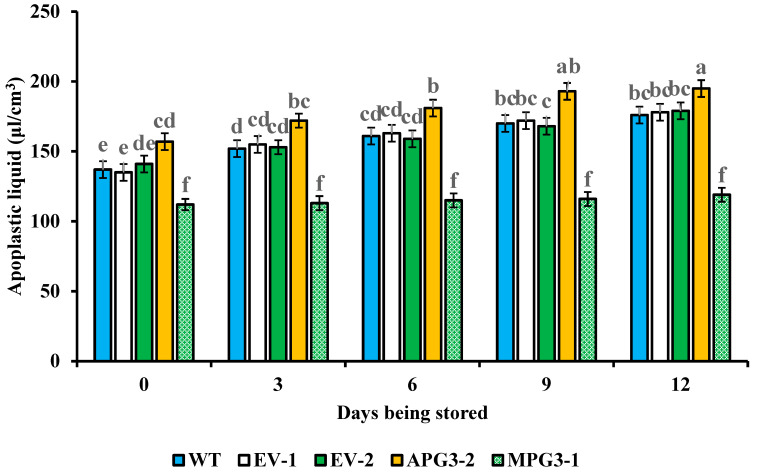
Apoplast liquid content in intercellular space of pineapple fruits. Different significance between treatment was shown in lowercase letters. All of the samples were determined three times and the average values were used. EV-1 represented pineapple transformed with empty vector when APG3-2 was constructed. EV-2 represented the pineapples transformed with empty vector when MPG3-1 was constructed. WT represented Tainong17 pineapple.

**Figure 8:**
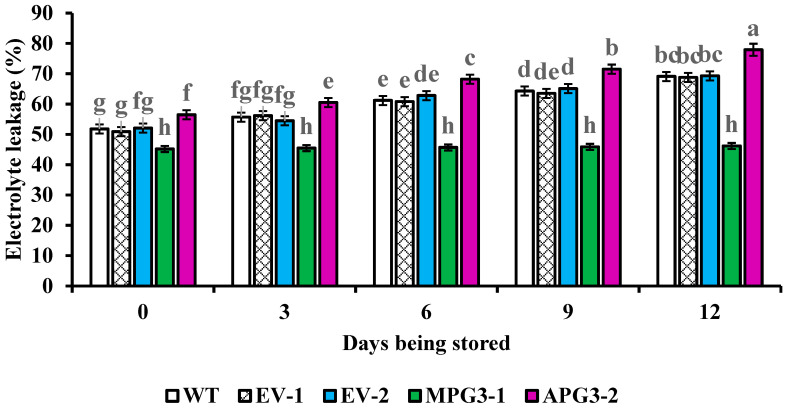
Electrolyte leakage of pineapple fruit flesh. Different significance between treatment was shown in lowercase letters. All of the samples were determined three times and the average values were used. EV-1 represented pineapple transformed with empty vector when APG3-2 was constructed. EV-2 represented the pineapples transformed with empty vector when MPG3-1 was constructed. WT represented Tainong17 pineapple.

To study whether cell inclusion leaks into the apoplast when pineapple fruit softens, the liquid content in the apoplast and electrolyte leakage of pulp were measured. The results showed that the liquid content in the apoplast of APG3-2 fruit was higher than those in MPG3-1, WT, EV-1 and EV-2 (Figure 7). The liquid content in the apoplast of MPG3-1 was lower than that of WT, EV-1 and EV-2 (Figure 7). No difference among the liquid content in the apoplast of WT, EV-1 and EV-2 was found. Similar results were also found in electrolyte leakage (Figure 8). For each line, the liquid content in the apoplast and electrolyte leakage increased with the softening process of pineapple fruit. These demonstrated that the expression of *AcoPG3* had a close relationship with the liquid content in the apoplast and electrolyte leakage in pineapple pulp. Mutating *AcoPG3* decreases liquid content in apoplast and electrolyte leakage. The shelf life of pineapple fruits can be extended by mutating *AcoPG3*.

## 4. Discussion

Pineapple fruits in China are mainly sold domestically. Only 5860 tons of pineapple fruits was sold to other countries in 2019. The exported pineapple fruits accounted for only about 0.34% of the total yield, according to China Customs data. The reason is that pineapple fruits cannot be stored for enough time to export to other countries.

Increasing activities of endogenous hydrolases are the main cause for fruit softening [3]. Polygalacturonase, PME, GAL, XET and CX are the main endogenous hydrolases in pineapple fruit [9]. During pineapple fruit ripening, polygalacturonase activity was much higher than that of PME, GAL, XET and CX. Polygalacturonase activity was positively correlated with the softening process of pineapple fruit. The enhancement of polygalacturonase activity is the main reason for the softening of pineapple fruit.

Pectin is a main component in the plant cell wall. Pectin can be degraded by polygalacturonase. After rice was transformed with polygalacturonase gene *OsPG2*, the pectin content in the root elongation zone of the transformed plant was less than that in the wild type [13]. The adhesion of the abscission zone in the petiole of apple tree transformed with *MdPG1* was weaker than that of the tree transformed with a net vector [14]. When the expression of *FaPG1* or *FaPG2* was inhibited, the HG pectin content and RG I pectin content in *FaPG1*-RNAi fruit and *FaPG2*-RNAi fruit were higher than those in control fruit [21]. In this research, polygalacturonase activity in APG3-2 fruit was the highest on the collection day. Polygalacturonase activity in APG3-2 fruit decreased with the storage time. Polygalacturonase activity in MPG3-1 fruit always increased, but the increasing velocity was very slow. Even on the 30th day after collection, the polygalacturonase activity of MPG3-1 was still less than that of WT on the collection day.

Polygalacturonase can degrade pectin on cellulose, a main component of the cell wall, that may change the component contents in the cell wall of flesh [34]. Suppressing the expression of the polygalacturonase gene in strawberry fruit resulted in increasing fruit firmness [23]. More HG and RG I pectin tightly bound to the cell wall were observed in the polygalacturonase-antisense strawberry line, suggesting that less pectin was dissocialized from the cell wall [21]. The abundance of methyl-esterified pectin from HG recognized was significantly higher in antisense polygalacturonase lines than in the control [21]. More pectin in antisense polygalacturonase lines was dissociated from the cell wall than the control [20]. In this research, the content of methyl-esterified pectin in the APG3-2 fruit cell wall was significantly less than that in MPG3-1, EV-1, EV-2 and WT fruit. The content of methyl-esterified pectin in the MPG3-1 fruit cell wall was more than that in EV-1, EV-2 and WT fruit. The contents of non-methyl-esterified HG and RG I in the cell wall of MPG3-1 fruit were higher than those in EV-1, EV-2 and WT fruit, respectively. Mutating *AcoPG3* inhibited the degradation of methyl-esterified pectin, non-methyl-esterified HG, galactose and RG I backbone.

When the cell wall is degraded by polygalacturonase, the cell membrane will expand outward, and the permeability of the cell membrane will increase [35]. Pores in the cell wall might be enlarged, and inclusion will leak into the apoplast. That will aggravate the softening process of pineapple fruit. The liquid content in the apoplast of APG3-2 pulp was higher than that in the apoplast of MPG3-1, WT, EV-1 and EV-2. The liquid content in the apoplast of MPG3-1 pulp was lower than that of WT, EV-1 and EV-2 pulp. Similar results were also found in electrolyte leakage. Mutating *AcoPG3* decreases polygalacturonase activity and delays the degradation of the cell wall. The permeability of the cell membrane and pores in the cell wall will not be enlarged so quickly, and the softening of pineapple fruits will be delayed.

Downregulations of polygalacturonase genes were previously all performed through RNAi or VIGS [22,23]. However, these methods cannot completely knock down the expression of the targeted gene, as they only provide temporary or partial inhibition of gene function [36]. We identified the function of *AcoPG3* in this research by constructing mutants and completely inhibited its expression. The phenotypes of the mutants were more stable, and the genetic traits can be passed down to the offspring steadily. These mutants can be used as germplasms for breeding pineapple cultivars with extended storage capacity in the future.

Fruit softening has a close relationship with electrolyte leakage and the apoplast liquid content. Although the functions of some polygalacturonase genes have been studied through transgenic experiments, neither electrolyte leakage nor apoplast liquid content have been studied [13,14,18,20,21]. We found that the liquid content in the apoplast of APG3-2 fruit was higher than that in MPG3-1, WT, EV-1 and EV-2. The liquid content in the apoplast of MPG3-1 was lower than that of WT, EV-1 and EV-2. Similar results were also found in electrolyte leakage. Mutating *AcoPG3* decreases the liquid content in the apoplast and electrolyte leakage of pineapple pulp.

Generally, in this research, the polygalacturonase gene *AcoPG3* was found to be the major gene responsible for the softening of pineapple fruit. Fruit of *AcoPG3*-overexpressing pineapple begins to soften earlier than that transformed with a net vector. Fruit of the pineapple-*AcoPG3* mutant began to soften later than that transformed with a net vector. The *AcoPG3* mutation resulted in a decrease in polygalacturonase activity in pineapple fruit, decreasing the degradation of methyl-esterified pectin, non-methyl-esterified HG, galactan and RGI backbone. Consequently, the degradation of cell walls and fruit softening were deferred. Extending the shelf life of pineapple fruit can be achieved by mutating the *AcoPG3* gene. This is the first study reporting the mechanism underlying pineapple fruit softening. These data have potential utilization in breeding pineapple cultivars with long shelf life in the future.

## 5. Conclusions

The polygalacturonase gene *AcoPG3* was found to be the major gene responsible for the softening of pineapple fruit. Fruit of *AcoPG3*-overexpressing tomato (*Solanum lycopersicum* var. Jingfan 101) begins to soften 9 days earlier than that transformed with a net vector. Fruit of *AcoPG3*-overexpressing pineapple began to soften earlier than that transformed with a net vector. Fruit of the pineapple line in which *AcoPG3* was mutated began to soften later than that transformed with a net vector. *AcoPG3* mutation resulted in a decrease in polygalacturonase activity in pineapple fruit, decreasing the degradation of methyl-esterified pectin, non-methyl-esterified homogalacturonan, galactan and the backbone of rhamnogalacturonan-I. The degradation of cell walls and fruit softening were deferred. Extending the shelf life of pineapple fruit can be achieved by mutating the *AcoPG3* gene.

## Figures and Tables

**Table 1:** Hydrolase activities of endogenous hydrolases in pineapple fruit at different stages ((µg.gal.acid)/(mg protein.h)).

Period	PG	PME	GAL	XET	CX
I	11.5 ± 0.3	0.34 ± 0.03	0.12 ± 0.02	1.25 ± 0.03	1.29 ± 0.03
II	12.7 ± 0.4	0.36 ± 0.03	0.17 ± 0.03	1.42 ± 0.03	1.42 ± 0.04
III	14.9 ± 0.5	0.37 ± 0.04	0.22 ± 0.04	1.55 ± 0.04	1.02 ± 0.02
IV	14.2 ± 0.4	0.38 ± 0.04	0.21 ± 0.04	1.47 ± 0.03	1.42 ± 0.04
V	13.5 ± 0.3	0.36 ± 0.04	0.18 ± 0.02	1.18 ± 0.03	1.25 ± 0.03

Note: PG represented polygalacturonase. PME represented pectinesterase. GAL represented β-galactosidas. XET represented Xyloglucan endotransglycosylase. CX represented cellulase. I showed the 2nd day after collection. Green peel area was more than 95%. II showed the 4th day after collection. Green peel area was more 80–90% of the total surface area of the pineapple fruit. III was the 9th day after collection. Yellow area of the peel was 20–40% of the total surface area of the pineapple fruit. IV was the 11th day after collection. Yellow peel area was 60–90% of the total fruit surface area. V was the 14th day after collection, when all of the peel was yellow.

**Table 2:** Water-soluble pectin contents and fruit firmness in different stages after collection.

Period	WSP (mg/g)	Firmness (kg/cm^2^)
I	1 ± 0.2	22 ± 0.5
II	4 ± 0.3	13 ± 0.4
III	17 ± 0.5	12 ± 0.4
IV	17.5 ± 0.5	9 ± 0.3
V	19 ± 0.6	7 ± 0.3

Note: WSP represented water-soluble pectin content. I showed the 2nd day after collection. Green peel area was more than 95%. II showed the 4th day after collection. Green peel area was more 80–90% of the total surface area of the pineapple fruit. III was the 9th day after collection. Yellow area of the peel was 20–40% of the total surface area of the pineapple fruit. IV was the 11th day after collection. Yellow peel area was 60–90% of the total fruit surface area. V was the 14th day after collection, when all of the peel was yellow.

**Table 3:** Fruit firmness of pineapple lines (kg/cm^2^).

DAH	WT	EV-1	EV-2	MPG3-1	APG3-2
0	26 ± 2.0 ab	25 ± 2.1 ab	25.6 ± 2.3 ab	29 ± 2.5 a	20 ± 1.5 bc
3	21 ± 1.1 bc	22 ± 1.5 b	21.7 ± 1.8 bc	28 ± 2.3 ab	10 ± 1.5 d
6	13 ± 1.2 c	12.6 ± 1.7 cd	12.8 ± 1.5 cd	28 ± 2.2 ab	8 ± 1.4 de
9	12 ± 1.5 cd	12.3 ± 1.3 cd	11.8 ± 1.3 cd	28 ± 2.0 ab	5 ± 1.6 f
12	9 ± 1.0 de	9.5 ± 1.2 de	9.7 ± 1.0 de	27.5 ± 2.0 ab	2 ± 1.4 gh
15	7 ± 0.7 ef	8 ± 1.1 de	7.5 ± 0.8 e	27.3 ± 2.2 ab	1 ± 0.5 h
18	6 ± 0.7 ef	7 ± 0.8 ef	6.8 ± 0.8 ef	27 ± 2.5 ab	1 ± 0.4 h
21	5 ± 0.9 fg	6 ± 0.7 ef	5.7 ± 0.7 ef	26.8 ± 2.6 ab	1 ± 0.4 h
24	3.5 ± 0.6 fg	4 ± 0.7 fg	4.6 ± 0.6 fg	26.3 ± 2.3 ab	1 ± 0.4 h
27	2 ± 0.5 gh	2.7 ± 0.5 g	3 ± 0.6 fg	26 ± 2.0 ab	1 ± 0.6 h
30	0.7 ± 0.5 h	1.1 ± 0.4 h	1.6 ± 0.5 h	25.5 ± 1.8 ab	1 ± 0.5 h

Note: DAH means days after harvest. Difference significance between treatments were shown with lowercase letters at *p* < 0.05 level. EV-1 represented pineapple transformed with empty vector when APG3-2 was constructed. EV-2 represented the pineapples transformed with empty vector when MPG3-1 was constructed. WT represented Tainong17 pineapple.

**Table 4:** Polygalacturonase activities of pineapple lines ((µg.gal.acid)/(mg protein.h)).

DAH	WT	EV-1	EV-2	MPG3-1	APG3-2
0	11.5 ± 0.5 de	11.2 ± 0.5 e	11.7 ± 0.5 e	3.7 ± 0.3 j	17.2 ± 0.6 a
3	12.7 ± 0.5 cd	12.5 ± 0.5 d	12.9 ± 0.5 cd	4.2 ± 0.3 ij	16.8 ± 0.6 ab
6	14.2 ± 0.6 bc	14.1 ± 0.6 bc	14.5 ± 0.6 bc	4.5 ± 0.3 i	16.5 ± 0.7 ab
9	14.9 ± 0.7 bc	15.1 ± 0.6 ab	14.7 ± 0.6 bc	5.1 ± 0.4 hi	16.1 ± 0.6 ab
12	13.5 ± 0.5 cd	13.6 ± 0.5 c	13.2 ± 0.5 cd	5.3 ± 0.4 hi	15.6 ± 0.6 ab
15	13.2 ± 0.5 cd	13.3 ± 0.5 cd	13 ± 0.5 cd	5.7 ± 0.4 h	15 ± 0.6 b
18	13 ± 0.5 cd	13.2 ± 0.5 cd	13.2 ± 0.5 cd	6.2 ± 0.4 gh	14.3 ± 0.6 bc
21	12.5 ± 0.5 d	12.7 ± 0.5 cd	12.9 ± 0.5 cd	6.6 ± 0.4 g	13.1 ± 0.5 cd
24	12 ± 0.5 de	12.3 ± 0.4 de	12.5 ± 0.4 d	6.9 ± 0.4 fg	12.8 ± 0.5 cd
27	11.6 ± 0.4 de	11.8 ± 0.4 de	12 ± 0.4 de	7.3 ± 0.4 fg	12 ± 0.5 de
30	11 ± 0.4 e	11.5 ± 0.4 e	11.7 ± 0.4 de	7.6 ± 0.4 f	11.6 ± 0.5 de

Note: DAH means days after harvest. Difference significance between treatments were shown with lowercase letters at *p* < 0.05 level. EV-1 represented pineapple transformed with empty vector when APG3-2 was constructed. EV-2 represented the pineapples transformed with empty vector when MPG3-1 was constructed. WT represented Tainong17 pineapple.

**Table 5:** Relative abundance of cell wall epitopes recognized by various mAbs in cell wall fractions.

Fraction	Line	LM18	LM19	JIM7	LM5	RU1	RU2	LM15	LM25
Water	WT	20 ± 3	5 ± 1	30 ± 3	0	0	0	0	0
	EV-1	19 ± 3	5 ± 1	31 ± 3	0	0	0	0	0
	APG3-2	13 ± 2	2 ± 1	21 ± 2	0	0	0	0	0
	EV-2	21 ± 3	5 ± 1	29 ± 3	0	0	0	0	0
	MPG3-1	31 ± 4	8 ± 1	41 ± 4	0	0	0	0	0
Na_2_CO_3_	WT	21 ± 3	20 ± 3	0	10 ± 2	25 ± 3	11 ± 2	0	0
	EV-1	20 ± 3	20 ± 3	0	10 ± 2	24 ± 3	11 ± 2	0	0
	APG3-2	12 ± 2	7 ± 2	0	3 ± 1	10 ± 2	5 ± 1	0	0
	EV-2	22 ± 3	21 ± 3	0	10 ± 2	24 ± 3	12 ± 2	0	0
	MPG3-1	32 ± 4	31 ± 4	0	16 ± 3	36 ± 4	19 ± 3	0	0
KOH	WT	20 ± 3	20 ± 3	0	60 ± 4	40 ± 3	40 ± 3	40 ± 3	72 ± 6
	EV-1	21 ± 3	20 ± 3	0	59 ± 4	41 ± 3	40 ± 3	39 ± 3	71 ± 6
	APG3-2	15 ± 2	16 ± 2	0	21 ± 2	22 ± 2	25 ± 2	40 ± 3	76 ± 6
	EV-2	20 ± 3	21 ± 3	0	61 ± 4	42 ± 3	41 ± 3	43 ± 3	73 ± 5
	MPG3-1	26 ± 4	27 ± 3	0	72 ± 4	65 ± 5	52 ± 4	41 ± 3	73 ± 6
Cadoxen	WT	0	0	0	20 ± 3	0	0	30 ± 3	31 ± 3
	EV-1	0	0	0	21 ± 3	0	0	29 ± 3	32 ± 3
	APG3-2	0	0	0	12 ± 2	0	0	30 ± 3	35 ± 4
	EV-2	0	0	0	22 ± 3	0	0	31 ± 3	33 ± 4
	MPG3-1	0	0	0	35 ± 4	0	0	31 ± 4	33 ± 3

Note: Water, Na_2_CO_3_, KOH, Cadoxen are solvents when components were extracted from fruit cell wall. LM18, LM19 and so on were antibodies bought from PlantProbes (Leeds, UK). EV-1 represented pineapple transformed with empty vector when APG3-2 was constructed. EV-2 represented the pineapples transformed with empty vector when MPG3-1 was constructed. WT represented Tainong17 pineapple.

## Data Availability

All data are hereby available in the manuscript.
